# Detecting Risky Authentication Using the OpenID Connect Token Exchange Time

**DOI:** 10.3390/s23198256

**Published:** 2023-10-05

**Authors:** Alex Heunhe Han, Dong Hoon Lee

**Affiliations:** School of Cybersecurity, Korea University, Seoul 02841, Republic of Korea; alexhan@korea.ac.kr

**Keywords:** risk-based authentication, OIDC protocol, identity and access management

## Abstract

With the rise in sophisticated cyber threats, traditional authentication methods are no longer sufficient. Risk-based authentication (RBA) plays a critical role in the context of the zero trust framework—a paradigm shift that assumes no trust within or outside the network. This research introduces a novel proposal as its core: utilization of the time required by OpenID Connect (OIDC) token exchanges as a new RBA feature. This innovative approach enables the detection of tunneled connections without any intervention from the user’s browser or device. By analyzing the duration of OIDC token exchanges, the system can identify any irregularities that may signify unauthorized access attempts. This approach not only improves upon existing RBA frameworks but is also in alignment with the broader movement toward intelligent and responsive security systems.

## 1. Introduction

While the COVID-19 pandemic is shifting into an endemic, the work-from-home culture initiated by almost every organization during the pandemic is expected to become the preferred work culture by employees, thus creating a new working environment. In March 2023, Gartner reported that 51% of U.S. knowledge workers hope to continue hybrid work, while 20% hope to work fully remote [1]. Companies have been pushing ahead with the application of Virtual Private Networks (VPNs), which allow employees to have direct access from the internet to the intranet, so that management information systems (MISs)—which were typically only accessible from inside the office—can be accessed from outside the company.

However, there are constant security threats, such as data exfiltration and the lateral movement of malicious codes, which occur while connected to the corporate network [2]. As a countermeasure, requiring users to install security posture applications at their endpoints is being considered as a prerequisite to accessing VPNs, and they will also be required to check its running status periodically. However, unfortunately, most employees—including high-level ones—see this as an inconvenience as endpoint security software usually conflict with other applications (e.g., anti-virus software, browsers, and so on) and operating systems.

As a solution, there is a movement that is pushing for the creation of digital workspaces. This involves migrating management information systems—which typically reside in a closed network—to a cloud network or even replacing it with a Software-as-a-Service (SaaS) applications. In this phase, enterprises are required to integrate with an enterprise identity and access management (IAM) system, which offers Single Sign-On (SSO) through a Security Assertion Markup Language (SAML) or an OpenID Connect (OIDC) protocol, as well as carries out the provisioning of identities through a System for Cross-domain Identity Management (SCIM) protocol. Through these steps, a high security level can be maintained by managing the access routes and detecting ongoing and potential threats.

This is also in alignment with the zero trust framework. Enterprises should constantly analyze and verify identities in every authentication process, as explained in the NIST SP 800-207 standard [3] and as part of the zero trust strategy, unveiled in July 2022 in the U.S. Department of Defense Reference Architecture [4].

Since its emergence, risk-based authentication (RBA) [5,6,7,8,9,10,11,12,13] has assumed a pivotal role within the zero trust framework, a paradigm shift that no longer assumes trust either within or outside of the network.

The crux of this research is the introduction of an innovative proposition: integration of the time required by OIDC token exchanges—which is a necessity in identity federation—as a novel feature of RBA. This groundbreaking approach allows for the identification of tunneled connections without necessitating intervention with a user’s browser or device.

Through a meticulous analysis of the duration of OIDC token exchanges, the system gains the ability to pinpoint irregularities that would be indicative of potential unauthorized access attempts. This method not only augments the existing RBA framework but also aligns seamlessly with the broader trend toward the implementation of intelligent and adaptive security systems.

The suggested method of utilizing OIDC token exchange times as a basis for RBA augmentation has the potential to allow for seamless integration with existing systems. This pragmatic approach bypasses the need for intrusive user interventions, thereby ensuring a user-friendly and efficient security protocol, and is detailed as follows:

1. Expansion of RBA coverage: While traditional RBA features concentrate on the first login (and, thus, encounter difficulties in precisely calculating risk), our approach ensures the collection of RBA features from every authentication, including Single Sign-On. Moreover, our approach completely aligns with the trend of the digital workspace, as every authentication that simultaneously happens in both public and private networks can be inspected;
2. Elaboration of user experience and security level: A complete server-based approach using OIDC token exchange logs eliminates any user intervention, which is common in traditional client- and script-based approaches. This serves to free the RBA system not only from the complaints of users who are anxious about their privacy but also from threats from attackers observing the RBA mechanisms, such as those seeking to avoid detection and aiming to gain access to valuable corporate assets;
3. Assurance of efficiency: Our approach to the nature of the authentication protocol itself does not require any additional solution or scripts to be deployed, which may ease the hesitation of companies to strengthen their authentication mechanism in order to achieve a zero trust framework. Furthermore, our approach has a high accuracy in detecting VPN-tunneling-based attacks and other security events in real-world scenarios.

The contributions of this research not only extend the boundaries of RBA but also underline its adaptability for the dynamic identification of potential security breaches. By harnessing time-based indicators, the system transcends traditional authentication approaches in order to proactively counteract emerging threats.

The remainder of this paper is organized as follows: Section 2 summarizes the primary contents of the paper and the characteristics of RBA, while Section 3 reviews the related literature. Section 4 details the proposed approach, while the experimental results are described in Section 5. A conclusion of the findings of this study is provided in Section 6. Abbreviations used in this study can be found in Appendix A.

## 2. Risk-Based Authentication

### 2.1. Concept and How it Works 

First, we provide an overview of what risk-based authentication (RBA) is and how it works. Risk-based authentication is a method for applying varying levels of stringency to authentication processes based on the likelihood of a security breach [7,8,9,10]. It is also often referred to as adaptive authentication. Its main goal is to enhance security measures for higher-risk scenarios while reducing potential user inconvenience during lower-risk situations.

**Figure 1:**
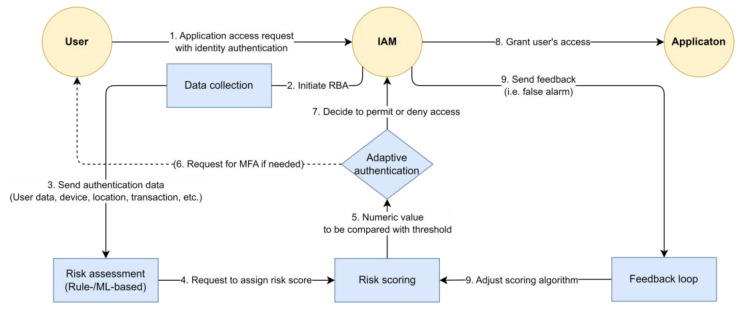
Risk-based authentication flow.

RBA adapts the type and depth of authentication needed based on the assessed risk level of a user, device, or transaction. The following Figure 1 provides a more detailed, step-by-step look at how it works:

(1) Data Collection: When a user attempts to access a system or perform a transaction, data related to that user, their device, their location, and the nature of the transaction are collected. This data could include the IP addresses, device type, operating system, browser type, geographical location, time of access, and so on. The user’s historical behavior data may also be collected, including typical login times, frequently used devices, and typical transaction patterns;
(2) Risk Assessment: The collected data are then analyzed with the risk-based authentication system, which uses algorithms and machine learning techniques to assess the risk level. This process might involve comparing the current behavior to the user’s historical behavior patterns, checking the IP address against databases of known malicious IPs, analyzing the geographical location, and more. The assessment process can incorporate a wide range of factors and is often customizable to a particular organization’s needs or risk tolerance;
(3) Risk Scoring: The system assigns a risk score based on the results of the risk assessment. This score is a numerical value that represents the perceived level of risk; for example, an unfamiliar device or an access attempt from a new location might increase the risk score;
(4) Adaptive Authentication: If the risk score falls below a certain threshold (indicating low risk), the user might be authenticated with just their username and password. However, if the risk score is above the threshold, the system will trigger additional authentication measures. This could include multi-factor authentication (MFA) methods, such as sending a one-time passcode to a user’s mobile device, requiring an answer to a security question, or using fingerprint or facial recognition;
(5) Feedback Loop: Most risk-based authentication systems have a feedback loop that allows them to learn from each access attempt; for example, if a user repeatedly logs in from a new location, and those logins are determined to be legitimate, the system might adjust its risk-scoring algorithm so that a lower risk score is assigned to logins from that location in the future.

By adjusting the authentication requirements based on the level of risk, RBA improves security while minimizing disruptions for the user. It provides an effective way of preventing unauthorized access and detecting fraudulent activities without causing any unnecessary inconvenience to legitimate users.

Many web services employ RBA to ensure the security of their platforms while optimizing the user experience [8]. For example, when a Google user tries to log into their account from a new device or location, Google might require additional verification steps, such as confirming the login attempt through a secondary email address or phone number. In addition, when a Facebook user logs in from a different geographical location or a new device, Facebook might prompt the user to verify their identity by identifying friends in tagged photos or sending a code to a trusted contact. Microsoft uses RBA for its cloud-based multi-factor authentication (MFA). This system assesses the risk of a login attempt based on factors such as unfamiliar sign-in properties or atypical travel, then prompts additional authentication if needed. 

### 2.2. RBA Features 

RBA collects various data [7,8,12] to evaluate a user’s login attempt. The data are then used to distinguish between normal and abnormal behaviors. The RBA system analyzes the data to calculate the risk score and determines whether to require additional authentication procedures based on the score. The 12 main data types collected are as follows:

(1) Login Location: This refers to the geographical location from which a user is attempting to access a system. This is often determined by the user’s IP address but can also involve GPS data from mobile devices. Unusual locations (e.g., access from a foreign country) can indicate potential fraud;
(2) IP Address: The IP address can provide information about the user’s internet service provider and geographical location. IP addresses can also be checked against lists of known malicious IPs. Frequent changes in IP address, or the use of anonymizing services such as VPNs, can increase the risk score;
(3) Device Information: Device information includes the type, make, and model of the device being used, the operating system, and the device’s unique identifiers. Access from an unrecognized or new device can be a sign of potential fraud;
(4) Login Time: The time at which a user typically accesses the system can be a factor in risk assessment. Logins occurring at unusual times (e.g., the middle of the night) might increase the risk score;
(5) Login Pattern: This refers to the user’s typical behavior when logging in, such as the frequency of logins, the time spent logged in, and the services accessed. Changes in these patterns can indicate potential fraud;
(6) Account Activity: Unusual account activity, such as a high number of transactions or changes to account settings, can be a sign of potential fraud. This can also include the type of transactions typically performed by the user;
(7) Keyboard and Mouse Behavior: Also known as behavioral biometrics, this involves patterns in the way that a user interacts with their device. This could include keystroke dynamics, mouse movements, and even touchscreen interactions. Deviations from established behavior can indicate that a different person is using the account;
(8) Previous Login History: This includes past instances of failed and successful logins, as well as the authentication methods used. Multiple recent failed login attempts can increase the risk score;
(9) Browser Fingerprint: This is a set of data that can uniquely identify the specific browser on a device, including the browser type and version, plugins installed, screen resolution, and other settings. This can help to identify whether a user is using a different or unusual browser, which could indicate fraud;
(10) Mobile Device Identifier: Unique identifiers for mobile devices, such as the international mobile equipment identity (IMEI) number, can be used to recognize trusted devices. Access from a new or different device can increase the risk score;
(11) Social Media (or Transaction) Activity: While not as commonly used due to privacy concerns and the complexity of data analysis, some systems may analyze social media activity for signs of potential fraud. This could include unusual activity or posts that contradict information known about the user;
(12) Round-Trip Time (RTT): This is the time that it takes for a data packet to travel from its source to a destination and back. High or varying RTTs can indicate network issues or the potential use of proxies or VPNs, which can be signs of fraudulent activity.

Nevertheless, user acceptability [8] must be considered when implementing the RBA system. The user’s acceptability may vary depending on the individual’s perception of security and personal information protection as well as how the data are collected. Some data types are collected from users more transparently and are recognized as important for security enhancement, while others can be considered as personal information infringement [9,10,11,12,13,14,15,16,17,18,19,20,21,22,23]. For this reason, it is important for the companies implementing RBA systems to provide transparency to their users and obtain consent if necessary.

There are multiple works that have identified the RTT as an effective feature for profiling and evaluating risks, as it does not need to expose its data collection and is thus free from privacy issues [7,8,24].

## 3. Related Works

### 3.1. Passive TCP Packet Analysis

TCP packets enable passive estimation of the round-trip time between the user and the server, as presented by Jay Aikat et al. [25]. Passive performance measurement tools mostly report RTT samples based only on the three-way TCP connection handshake. However, they may be biased when SYN/SYN-ACK packets are processed differently, as compared to regular TCP packets; for example, SYN/SYN-ACK packets might have to go through a middlebox or be delayed using a remote server before new connections are accepted. Thus, it is challenging to continuously monitor RTT in TCP traffic.

Xiaoqi Chen [26] addressed this limitation and suggested an improved approach that focuses on the data plane of the TCP packet using a multi-stage hash table, thus enabling continuous passive estimation. However, this method requires the deployment of a programmable network switch in front of the server, which implies financial or physical limits.

Gerad et al. [27] and Sical Lv et al. [28] suggested a machine-learning-based approach for VPN traffic identification, using various timing features derived from the TCP packet. But still, these methods heavily depend on deep packet inspection technology, which needs additional deployment of a deep packet inspection solution in the middle of a network path. 

The adoption of public cloud services has been accelerating, and most cloud service providers are hesitant to allow their clients to manipulate network switches or deploy their own network switch. For example, Amazon Web Services (AWS), which is one of the most popular public cloud service providers, supports measurement of the RTT. However, they focus on the RTT in order to be able to track the latencies of the cloud service itself—such as the latencies of the AWS region or the internet service provider—and not the latencies of connected individual users.

To summarize, analyzing the TCP packet supports passive and continuous estimation of the RTT without any user intervention or the risk of being exposed to the attacker. However, there is surely a limit to its utilization in the real world, as its deployment is not easy.

### 3.2. Active Web-Ping Response Time Measurement

Packet internet groper (Ping) is a computer network administration utility that helps to determine whether a particular IP address or domain is accessible on the computer network or not. Ping works by sending a packet to a provided address and then waiting for the reply while also measuring the round-trip time and reporting errors. 

There have been many studies trying to verify location using Ping. Katharina et al. [29] measured the connecting location by measuring Ping response from surrounding probes. Biran et al. [30] introduced a machine-learning approach to locate IP geolocation based on Ping response data. This Ping technology is being widely studied in the world of the Internet of Things to locate and monitor widely spread Internet of Things devices [31,32,33,34,35,36].

However, web browsers generally do not support Ping, thus Ping technology is not appropriate for a web-based environment. The client program must be deployed to the user device in order to send Ping and calculate Ping response time, which will bring lots of user inconvenience.

**Figure 2:**
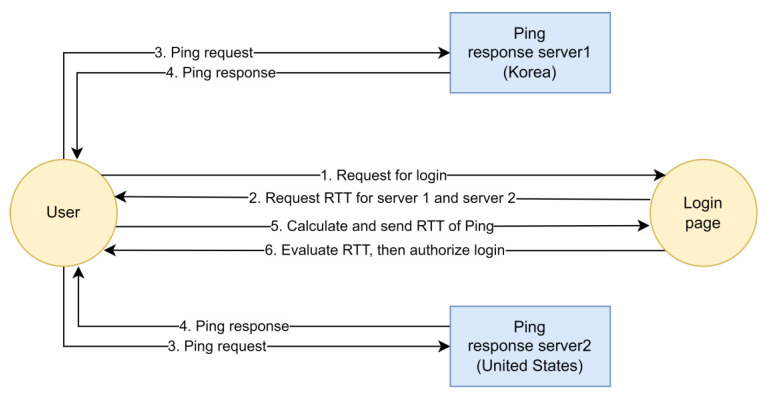
Measuring the RTT using the Client-to-Server web-Ping.

Instead, WebSocket technology—which is integrated into most online browsers at present—supports the active measurement of the RTT via Ping. Rivera et al. [37] proposed a client-based round-trip measurement method that sends web-Ping packets from the client side to nearby servers and authentication servers. Then, the duration of the web-Ping response is calculated. Figure 2 depicts their proposed method. 

In this approach, JavaScript (JS) must be sent to the user’s web browser and then executed. Thus, user intervention is required, and an attacker in the middle may modify the JS or send manipulated RTT measurement results. Additionally, as there must be a trigger point for the web-Ping, the RTT measurement point is limited to the first login page.

**Figure 3:**
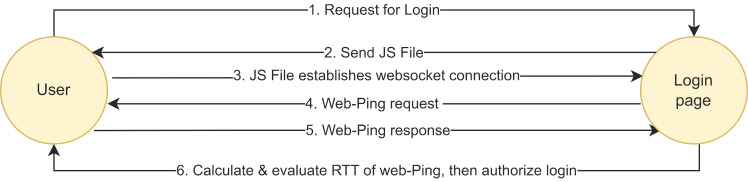
Measuring the RTT using the Server-to-Client web-Ping.

Wiefling et al. [24] presented a round-trip measurement method that is less client-based by requiring the client to establish a web socket session via a JS file, which is downloaded to the client web browser when the server login page is accessed. Then, the server sends a web-Ping to the client and waits for a response. Figure 3 depicts their proposed method. 

However, the JS file still needs to be sent to the client and operated. Hence, user intervention and the possibility of forgery cannot be eliminated. This approach suggests sending multiple web-Pings to measure the RTT, although the target is still limited to the login page (where the JS file is sent to the user’s web browser).

To summarize, active web-Ping measurement requires user intervention and can be easily observed [38], therefore opening it up to the possibility of being manipulated [39]. In addition, it has definite limits regarding continuous monitoring, and so, the RTT cannot be measured in the case of re-authentication (SSO) after the first login.

### 3.3. Wi-Fi Round-Trip Time Measurement 

Fine Time Measurement (FTM) protocol, which is part of the WIFI 802.11 mc protocol, enables a pair of Wi-Fi cards to estimate distance between them. This allows a device to measure the distance to a Wi-Fi Access Point (WAP) in a bi-directional communication process. The user terminal initiates this process, and the WAP responds to the query, which eventually results in an estimation of the distance through the round-trip time.

Orfanos, Manos, et al. evaluated the effectiveness of Wi-Fi round-trip time to solve indoor localization problems [40]. Garcia-fernandez, Miquel, et al. [41] presented automated Wi-Fi access point estimation using Wi-Fi round-trip time. Satiaseelan Selvan et al. [42] implemented a risk-based model to detect confidentiality-based attacks to wireless fog-IoT environments.

This Wi-Fi round-trip time measurement is a powerful way to locate Wi-Fi users who are using a corporate wireless connection but cannot cover remote workers accessing a corporate system via wired public internet connection.

### 3.4. Authentication Path Analysis

With a perspective on the round-trip itself, the active directory authentication logs of internal endpoints and servers inside a corporate network are collected to identify the authentication paths, in order to be able to calculate the abnormality of paths and detect the horizontal movements performed by attackers [43]. 

There is no chance for the attackers to sense and avoid this detection system, as the data are directly collected from the authentication server’s active directory, which completely operates as server-side logic.

However, there is a clear limit to the centralized collection of authentication logs generated from each device in a public internet environment, given that there are many stakeholders, including various internet service providers. Especially in the case of attackers with a VPN-tunneled connection, there are clear limits to the acquisition of logs from VPN service providers who advertise anonymity.

### 3.5. Comparison

Table 1 provides a comparison of the various methods introduced so far in this paper. 

“Continuously measurable” refers to whether the proposed method collects round-trip data not only at the first login step (which prompts credential input) but also at further logins using a pre-authenticated session acquired through the first login. As the RTT can be affected by temporary changes in network traffic or the user device environment, it is much better to collect the RTT continuously. 

“Network coverage” refers to whether the proposed method can be used not only in a private corporate network but also in a public internet environment. As protected assets are being moved to a public cloud, it is important to cover not only private networks but also public networks.

“Deploy huddles” refers to whether certain efforts are required to achieve deployment. There are physical limits if a certain solution needs to be deployed in front of the server, and the budget will also increase when a commercial solution is considered. Furthermore, if any of the files (including the script) are sent to user’s web browser, there may be resistance from the user or some anti-virus program might judge it as malware and block it.

“User intervention” refers to whether intervention is needed from the client side. Passive measurement from the server side (e.g., using packet dump or log analysis) does not require any intervention, in contrast to the active measurement from the client side. 

“Attacker Observable” refers to whether the attacker can sense that their RTT is being monitored. Web debugging tools [38] can support attackers in capturing the scripts sent to the web browser and manipulating them to avoid detection.

Considering these points of comparison, we propose a new approach that uses the round-trip time of the OIDC authorization code as a new RBA feature, ensuring continuous measurement without network coverage limitations while not consuming client resources, thus filtering out attackers trying to observe the RBA features.

## 4. Proposed Approach

### 4.1. Identity Federation

Identity and access management (IAM) is a framework that manages digital identities and access permissions in an organization. IAM ensures that the right individuals have access to the right resources at the right times and for the right reasons.

By controlling who has access to what, IAM helps in reducing the risk of unauthorized access to sensitive information. In addition, as many industries have regulatory requirements that mandate control over who can access specific data, IAM helps in meeting those requirements. Moreover, by automating and streamlining the access control process, IAM makes it easier for users to obtain the resources they need and for administrators to manage those permissions.

Identity federation—a subset of IAM—extends this in order to allow interoperable access across different organizational boundaries. It enables the linking and use of a single identity across multiple independent systems or organizations. Through the federation, a user can log in once (SSO), then access various systems without having to re-authenticate. This also drastically reduces the need for locally provisioned accounts and provides IAM administrators with more centralized visibility and control over accounts [44].

Commonly used standard protocols for identity federation include Security Assertion Markup Language (SAML), Open Authentication (OAuth), and OpenID Connect (OIDC).

### 4.2. OIDC Protocol

Our approach focuses on OIDC, an identity layer built on top of the OAuth 2.0 protocol that allows client applications to verify the identity of end-users based on the authentication performed with an authorization server.

OIDC allows Single Sign-On (SSO) to all types of client applications—including web-based, mobile, and JavaScript clients—to enable them to request and receive information about authenticated sessions and end-users. Services that support OIDC can also utilize optional functions such as ID data encryption, OpenID provider search, and session management.

**Figure 4:**
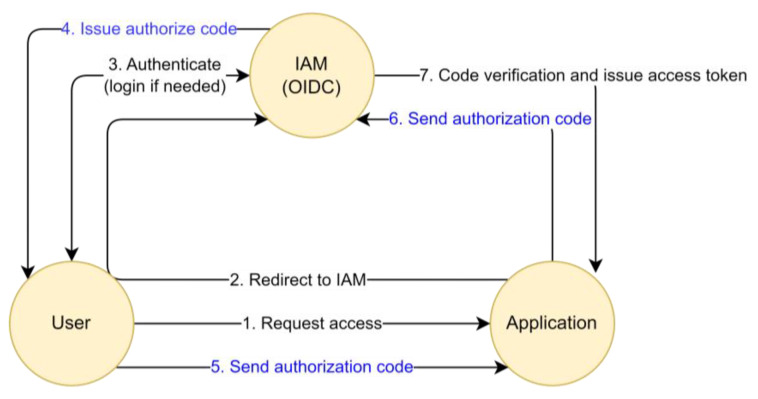
IAM based on the OIDC protocol.

The OIDC flow is depicted in Figure 4 below.

The protocol is detailed as follows:

(1) A user wants to use a service, usually a web-based one that is provided by an application. The application requires an account to be able to provide the service. The user issues a request to the application to be able to access their identity information, which is stored on the IAM server;
(2) To access the user’s information on the IAM server, the application requires authorization from the user. In order to prevent a replay attack of the authorization code, the application creates a challenge code and a verification code using nonce. Then, the application redirects the user’s web browser to an IAM server along with the challenge code and the code challenge method;
(3) The IAM server prompts the user to login using their credentials if there is no pre-authenticated session. The session key is stored in the user’s web browser once authenticated; this step is bypassed in the case of a Single Sign-On (SSO);
(4) The IAM server creates an authorization code and delivers it to the user with a callback URL that has been pre-registered on the IAM server by the application. The IAM server also stores the received challenge code and the code challenge method for further verification;
(5) The user sends the received authorization code to the accessing application through a received callback URL;
(6) The application requests the IAM server to change the authorization code into an access token. The application also appends the verification code to the request;
(7) The IAM server verifies the received verification code with the stored challenge code and the code challenge method. In addition, it verifies the authorization code and then issues the access token, sending the requested identity information as well. Then, the application checks the user’s identity information (which the IAM server provides) and permits the user’s access to their service. The challenge code is revoked to prevent a replay attack.

The OIDC is a modern, standard SSO protocol that has significant advantages in terms of security, compared to SAML (Security Assessment Markup Language), as recommended by the U.S. Department of Defense [44]. OIDC uses “authorization code flow” (see steps 4–6 above) to enforce security. The process of changing the code into tokens in the back channel prevents tokens from moving through the user’s browser, reducing the possibility of exposing specific credentials through direct communication between the application and the authentication server. In addition, security increases as verification procedures for client applications are added in the process of exchanging tokens with client applications on authentication servers.

To summarize, OIDC supports enhanced security in identity federation through its core “authorization code flow” mechanism. 

### 4.3. OIDC Token Exchange Time as RBA Feature

**Figure 5:**
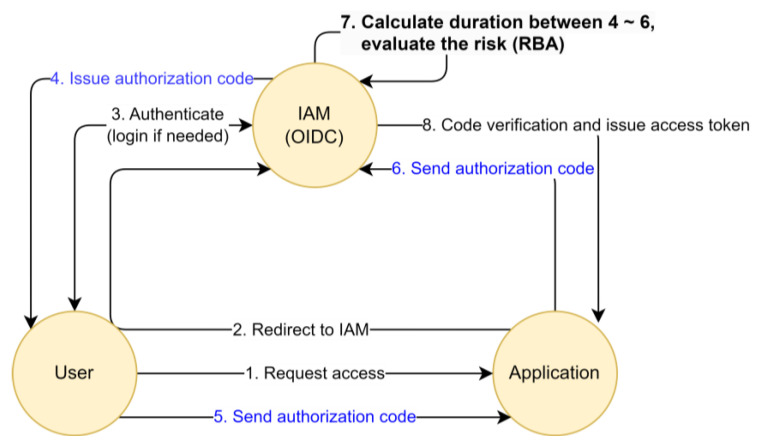
Calculating the TXT as an RBA feature.

In this paper, we define the round-trip time in the OIDC “authorization code flow” core mechanism as the token exchange time (TXT), and we use TXT as an RBA feature. Figure 5 depicts the usage of TXT as a new RBA feature. 

TXT is the time it takes for the authorization code to leave the OIDC-supporting IAM server (4), be redirected to the client application via the user browser (5), and then return to the IAM server again (6), in order to be exchanged for an access token. TXT can be calculated by subtracting the time in (4) from the time in (6). The calculated TXT can be used to evaluate risk (7) when issuing access (8).

An authorization code flow occurs regardless of the connected network location or pre-authenticated status. This enables the TXT to be generally measured without spatial and/or timing limits.

TXT can be collected passively from OIDC-supporting IAM server applications without any interactions with the user’s web browser. As OIDC server applications generally support authorization code flow, logging, collecting, and analyzing audit logs of the OIDC server can be an easy way to obtain TXT data. When we set up our test environment using a WSo2 identity server [45]—one of the most popular open-source OIDC server applications—we were able to identify the start and end of the token exchange. The audit log indicated when the authorization code request for the authenticated user was received, as well as when the user’s authorization code came back from the client application for access token exchange. 

Table 2 below shows example logs from a WSo2 identity server installed in our test environment, located in Korea.

The first row in Table 2 indicates that the IAM server issues an authorization code *(ResponseType: code)*—see (4) in Figure 4—to the user (*user: example000055@example.company*) who wants to access *(Authorization Request received for user)* the application (*ClientID: 2_Rc5iEXGeppgMmLNwH4ogVBqNIa*) and prompts the authorization code through the application using a callback URL (*RequestedcallbackURI:* https://stg.sid.sam.net/qms/exam). The event time (*10 July 2023 10:19:05,527*) can be used as the starting point of token exchange. 

The second row in Table 2 indicates that the IAM server receives the issued authorization code *(Found Authorization Code for Client: 2_Rc5iEXGeppgMmLNwH4ogVBqNIa)*, which is passed through the user and application—see (6) in Figure 4. The IAM server recognizes this authorization code was received for the token exchange, as part of OIDC *(scope:openid),* and matches the owner of the authorization code *(authorized user: example000055@example.company)*. The event time (*10 July 2023 10:19:06,211*) can be used as the finish line of the token exchange. 

Thus, the time gap (685 msec) can be calculated as the TXT of the user *(example0005@example.company)* who is accessing the application *(ClientID: 2_Rc5iEXGeppgMmLNwH4ogVBqNIa).*

### 4.4. Profiling and Anomaly Detection Design

In designing the TXT profiling approach for anomaly detection, we have to point out that the authorization code travels not only between the OIDC server and the user browser but also to the client application before being changed into a token. 

The time it takes for the authorization code to travel from the IAM server to the application via a user will vary based on the networking environment, thus influencing the overall TXT time. The trip time from each application to the IAM server is not affected by the user environment and is usually constant since an enterprise requires applications with a stabilized connection environment.

**Figure 6:**
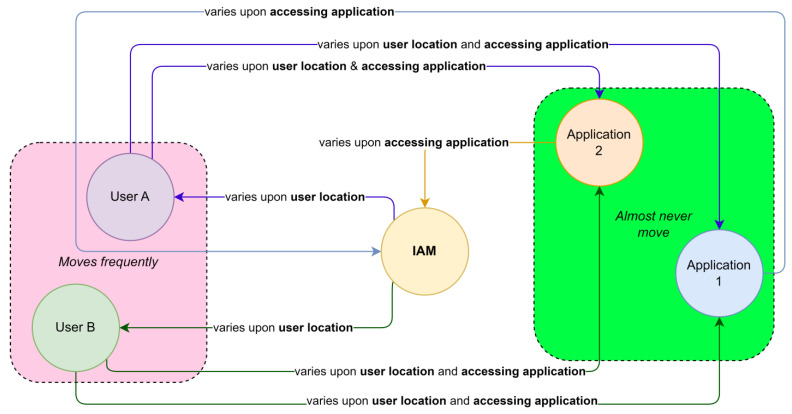
Authorization code flow when multiple users access multiple applications.

Figure 6 describes how overall TXT is influenced upon user location and accessing applications. 

**Figure 7:**
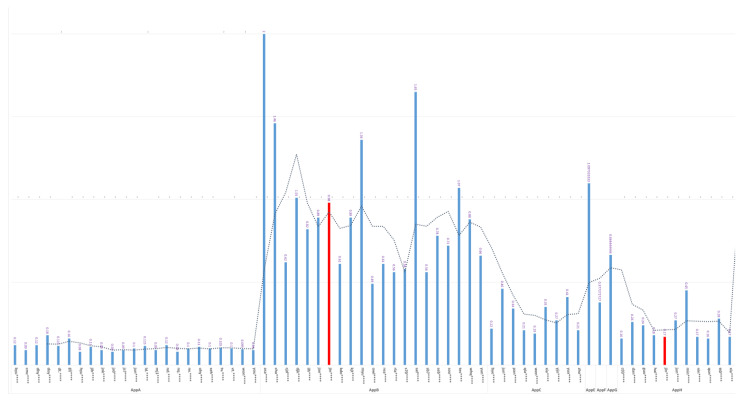
Example of profiled TXT per application and user.

Based on the above analysis, we decided to profile the TXT per user for each client application. An example of user TXT profiles per application is shown in Figure 7 below. The detailed query used to produce this profile is provided in Appendix B.

The red-colored bar indicates the average TXT value of the user jin**** when accessing App B (0.98 s) and App H (0.17 s). The TXT values are different from each other, even though the token exchange occurred from the same user. 

If a user’s TXT value per application has obvious changes—for example, if TXT spikes to 0.58sec when the user jin**** accesses App H, this means that the user’s physical location has changed, resulting in changes in the time required by the authorization code to travel from the IAM server to the accessing application, passing through the user’s location. 

## 5. Experiments

### 5.1. Design

Experiments were designed to verify the following observations:

(1) TXT changes when the user connects through a VPN:
  Using a VPN is a popular way for the attackers to hide their IP and avoid an IP-based detection system. Thus, we attempted to determine whether the TXT differs even though the IP does not change, as a proxy may hide an attacker’s real IP;
(2) TXT changes when a user connects through a mobile hot-spot:
  At present, many companies strongly recommend that remote workers access the corporate MIS system only from designated remote workplaces. In other words, connecting through the use of mobile hot-spots from public places (e.g., cafes or libraries) is not recommended as there is a risk of unintended information leakage via a shoulder surfing attack. Thus, we tested the change in TXT in the case of hot-spot users;
(3) This approach works well in a real environment as well:
  Our approach was successful in the lab test environment and, so, we tested the efficiency of TXT-based detection in a real environment.

Table 3 details how the experimental environments were prepared.

### 5.2. TXT Changes When User Connects via VPN 

We prepared multiple user PCs from all over the world using the Google Cloud Platform (GCP) and installed the Surfshark VPN. For each PC, we established a connection with the Korean VPN server, then sent 10 requests for the OIDC-based authentication to gain access to the mock web application. We also made 50 authentication requests from a PC located in the Korea region, without being VPN tunneled.

Table 4 provides the experimental results.

**Figure 8:**
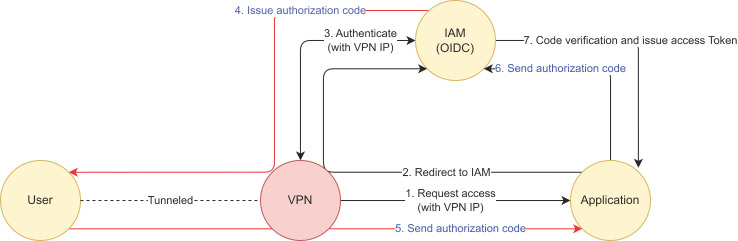
Authorization code flow under VPN tunneling environment.

VPN tunneling deceived the source IP of authentication request as 61.255.174.x, which is the IP of the Korean VPN server. But since the authorization code from the IAM server needs to travel over the user’s browser, who is hiding behind VPN server, the latency for network hop is added and showed much higher TXT value than a non-VPN connection. Figure 8 describes how the authorization code flow changes under a VPN tunneling environment.

A comparison of the VPN-tunneled TXT average and minimum values with the plain average TXT value (119 ms) is given in Table 5.

To summarize, we observed that VPN users presented higher TXT values (over 1.77 ratio) than plain users, with the TXT varying per global region even though the IP was hidden behind the same VPN IP server address. This proves the possibility of detecting a VPN-tunneled connection through the use of TXT.

### 5.3. TXT Changes when User Connects via Hot-Spot 

We prepared a PC connected to the public internet through a home internet service provider (Korea Telecom). Likewise, we prepared an Android mobile phone (Galaxy Flip2) coupled with a mobile carrier (LG Uplus), which supports a mobile hot-spot that enables a connected device to access the public internet through a mobile carrier.

We measured the TXT from the PC by requesting OIDC-based authentication 10 times using the same mock web application without a mobile hot-spot connection, and we measured again with a mobile hot-spot connection another 10 times. We also tested the TXT from the mobile phone using a mobile web browser.

Table 6 provides the experimental results.

**Figure 9:**
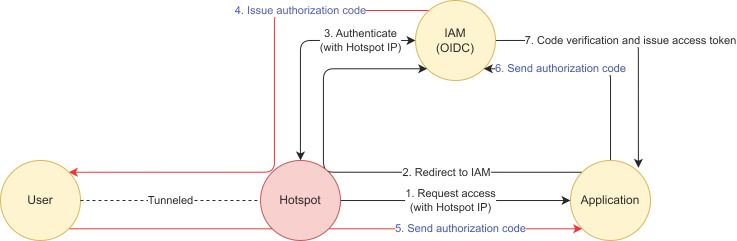
Authorization code flow under hot-spot tunneling environment.

Hot-spot connection shows a higher TXT value than a non-hot-spot connection, like the VPN connection experiment result in the previous section. The network hop between the hot-spot and user device increases TXT value, as described in Figure 9 below.

A comparison of the hot-spot-tunneled TXT average and minimum values with the plain average TXT values (home internet: 84 ms and mobile internet: 168 ms) is given in Table 7.

We observed that hot-spot users presented higher TXT values (with a ratio of over 1.67) than plain PC users. Hot-spot users still displayed higher TXT values (average ratio 1.15) than a mobile user, but sometimes it was not possible for TXT to distinguish hot-spot users from plain mobile users. In contrast to VPN users who usually transit to a remote VPN server, usually located outside of their country, hot-spot users often connect to their own, possessing a mobile device in close physical distance; this makes it hard to distinguish hot-spot users from mobile users.

However, when we matched these to the browser user-agent information—which can easily be extracted from the web log—we were able to determine the device information, as shown in Table 8.

To summarize, we observed that hot-spot users presented higher TXT values (with a ratio of over 1.67) than plain users, thus proving the feasibility of TXT in detecting hot-spot users.

### 5.4. Observed Findings in Real Traffic (Hot-Spot User, Account Sharing)

For the experiment conducted in a real environment, we collected OIDC logs from the stage IAM server (112.107.*.*, IP address is masked with *) of a large IT service company, which is the IT arm of a top global brand. In July of 2023, there were 4561 OIDC token exchanges for 153 users accessing 18 applications. The applications were accessible via public internet, as it was developed to service remote workers.

For every application, we profiled the average TXT of users over the last 3 months and watched whether there were any authentication requests exceeding the average by 67%, based on the prior experimental results (Section 5.2 and Section 5.3).

From the real traffic, there were no attacks using VPNs. Nevertheless, meaningful cases were observed from the perspective of account security.

When we analyzed real traffic, it was possible to observe the Chrome browser on a Windows 10 PC occasionally, connecting via a mobile hot-spot in a public place. In the case of a general connection through home or office internet, the TXT time was low due to the fast and stable network connection. In contrast, the TXT time increased when access was made through a mobile hot-spot, which was detected as risky authentication, as indicated in Table 9.

This case was detected when the user jae.*.k** (user id is masked with *) accessed the specific application with an IP hosted by a public cloud service (123.37.*.*, IP address is masked with *) and through mobile hot-spot tunneling. The logged IP was allocated to a mobile carrier when a search was performed using the whois.com site [47]; however, as we previously revealed in Section 5.3, the collected user-agent data indicated the use of a Windows 10 PC as the connected device environment, as described in Table 10. 

Another interesting point observed was the possibility of detecting account sharing activity. When the account of jae.*.k**—who is a project manager mainly working at the HQ—was shared to project members working in remote working spaces, the TXT time showed various changes. Compared to the TXT of the office environment (1151 ms), the TXT was occasionally delayed by more than 35% (1586 ms). 

To summarize, we were able to observe that the detection of risky authentication using the TXT time worked well in a real-world scenario.

## 6. Conclusions

Our approach gives a much easier option for the enterprises seeking to upgrade their authentication system, making it more secure and more seamless, and integrate logs from their present authentication server without purchasing an expensive traffic analyzer or distributing Javascript to privacy-concerning users. This simple but novel approach will effectively block attackers’ attempts to avoid detection; their activity will be tracked silently from complete server-side and continuously whenever they trigger the login or Single Sign-On. 

Measuring the time taken for OIDC token exchange enables the highlight of a VPN- and hot-spot-tunneled authentication request, which avoids IP based detection, as proven in the previous section. This is because the network latency between the user and the tunneling device is added, presenting increased round-trip time. 

A user may change their network environment in a legitimate way, such as changing their internet service provider or moving to another country; however, this may trigger a false alarm, the same as other round-trip based detection approaches. An automated update of the detection threshold to reflect the user’s trends [48] and prompting the user to check whether it was legitimate authentication, via multi-factor authentication, would be effective ways of completing an adaptive detection system and minimizing false alarms. 

Moreover, a correlation analysis using GeoIP or browser fingerprinting features (e.g., user-agent information as shown in the previous section) may help to reduce false alarms, giving the security administrator a clear view to be able to identify risky authentication. In future research, we plan to determine other useful features that can be coupled with token exchange time.

In the context of the zero trust framework, which hinges on the principle of distrust, the significance of RBA becomes amplified. In response to the intricate landscape of cyber threats, consideration of the time factor in OIDC token exchanges introduces a layer of sophistication to the RBA mechanism.

With the realm of cybersecurity continuously evolving, the proposed time-based RBA enhancement aligns harmoniously with the trend of intelligent and responsive security systems. Its adaptability sets a precedent for future security methodologies that prioritize accurate threat detection and prevention.

In conclusion, the incorporation of time-based OIDC token exchange analysis within RBA embodies a forward-looking approach to security enhancement. By embracing the principles of zero trust and leveraging advanced authentication mechanisms, this research contributes to the ongoing battle against evolving cyber threats.

Since OIDC supports compatibility with mobile apps and provides enhanced security features that are essential for companies preparing digital workspaces based on the zero trust framework, we expect that OIDC-supported corporate SaaS apps will gradually be developed. However, many SaaS apps still only support SAML, which is a much older protocol than OIDC. Our research focused on the nature of the OIDC protocol, but we will also look into the SAML protocol to find potential RBA features. 

## Figures and Tables

**Table 1:** Comparison of various methods.

Methods	Continuously Measurable	Network Coverage	Deploy Huddles	User Intervention	Attacker Observable
Passive TCP Packet Analysis [25,26,27,28]	First Login, SSO	Public, Private	High (Traffic Analyzer)	No	No
Active Ping Response Time [29,30,31,32,33,34,35,36]	First Login Only	Public, Private	High (Agent)	Yes	Yes
Client-to-Server Active Web-Ping Response Time [37]	First Login Only	Public, Private	Medium (Web browser Script)	Yes	Yes
Server-to-Client Active Web-Ping Response Time [24]	First Login Only	Public, Private	Medium (Web browser Script)	Yes	Yes
Wi-Fi Round-Trip Time [40,41,42]	First Login, SSO	Private Only	Low (Wireless Lancard)	No	No
Authentication Path Analysis [43]	First Login, SSO	Private Only	Low (Server Logging)	No	No
Token Exchange Time (Proposed Method)	First Login, SSO	Public, Private	Low (Server Logging)	No	No

**Table 2:** Example token exchange start and finish logs.

Step	WSo2 Log Pattern	Sample Logs
Start (4)	Authorization Request Received for User	[2023-07-10 10:19:05,527] [489456ae-db04-498d-9deb-c99f0c011759] DEBUG{org.wso2.carbon.identity.oauth2.OAuth2Service}- Authorization Request received for user: EXAMPLE.COMPANY.USERSTORE/example000055@example.company, ClientID:2_Rc5iEXGeppgMmfLNwH4ogVBqNIa,AuthorizationResponseType:code, RequestedcallbackURI: https://stg.sid.sam.net/qms/exam
Finish (6)	Found Authorization Code for Client	[2023-07-10 10:19:06,211] [9bccf412-1368-4b5c-a21b-a296a6a96ddd] DEBUG {org.wso2.carbon.identity.oauth2.token.handlers.grant.AuthorizationCodeGrantHandler}– Found Authorization Code for Client: 2_Rc5iEXGeppgMmLNwH4ogVBqNIa, authorized user: EXAMPLE.COMPANY.USERSTORE/example000055@example.company, scope: openid

**Table 3:** Specifications for the prepared experiment environments.

Spec	IAM Server	VPN Test PC	Hot-spot Test PC	Hot-spot Mobile
Location	East Asia (Korea)	America (North/South), Europe, Middle East, Australia, Asia (South/East)	East Asia (Korea)	East Asia (Korea)
OS	Redhat Linux	Debian GNU/Linux	Windows 11 Pro	Android
CPU	6 vCore	1 vCore	4 Core	SM-F707N (Galaxy Z Flip)
MEM	48 GB	4 GB	16 GB	
Disk	SSD 600 GB	Persistent Disk 10 GB	SSD 512 GB	
Software	WSo2 Identity Server [45], Mock Web application [46], MariaDB	Chrome, Surfshark VPN	Chrome	Chrome (Mobile)

**Table 4:** TXT values of VPN and non-VPN users.

GCP Region	GCP Public IP	VPN Tunneled IP	TXT Average	TXT Max	TXT Min
australia- southeast1-b	34.116.113.234 (Australia)	61.255.174.30 (Korea)	237	294	216
europe- west4-a	34.32.163.9 (Netherlands)	61.255.174.211 (Korea)	510	1225	332
asia- south1-c	35.244.21.199 (India)	61.255.174.30 (Korea)	228	252	210
me- central1-a	34.18.22.213 (Qatar)	61.255.174.211 (Korea)	369	434	344
southamerica -east1-b	35.198.32.210 (Brazil)	61.255.174.254 (Korea)	449	1199	359
us- west4-b	34.125.209.117 (U.S.A.)	61.255.174.254 (Korea)	258	341	233
asia- northeast3-a	34.64.112.18 (Korea)	No VPN	119	201	97

**Table 5:** Comparison of TXT value statistics between VPN and non-VPN users.

GCP Region	Average TXT Increase	Average TXT Increase Ratio	Min TXT Increase	Min TXT Increase Ratio
australia- southeast1-b	118	1.99	97	1.82
europe- west4-a	391	4.30	213	2.80
asia- south1-c	109	1.92	91	1.77
me- central1-a	251	3.11	225	2.90
southamerica-east1-b	331	3.79	240	3.03
us- west4-b	140	2.18	114	1.96

**Table 6:** TXT values for hot-spot and non-hot-spot users.

Device	Connection	IP	TXT Average	TXT Max	TXT Min
PC	Hot-Spot	106.101.65.217 (LG Uplus)	194	303	141
PC	Home Internet	222.107.198.110 (Korea Telecom)	84	138	68
Mobile	Mobile Internet	106.101.65.168 (LG Uplus)	168	380	120

**Table 7:** Comparison of TXT value statistics between hot-spot and non-hot-spot users.

Type	TXT Increase from Home Internet	Ratio of TXT Increase from Home Internet	TXT Increase from Mobile Internet	Ratio of TXT Increase from Mobile User
Average TXT (194ms)	110	2.30	26	1.15
Min TXT (141ms)	57	1.67	−27	0.84

**Table 8:** Comparison of collected user-agent data.

Device	Connection	User-Agent Data
PC	Hot-Spot	Mozilla/5.0 (Windows NT 10.0; Win64; x64) AppleWebKit/537.36 (KHTML, like Gecko) Chrome/116.0.0.0 Safari/537.3
PC	Home Internet	
Mobile	Mobile Internet	Mozilla/5.0 (Linux; Android 10; K) AppleWebKit/537.36 (KHTML, like Gecko) Chrome/115.0.0.0 Mobile Safari/537.36

**Table 9:** Detection of hot-spot users.

Result	IP (Masked with *)	Average TXT	Date	Count	ISP (Masked with *)	Note
Profiled (Normal)	203.244.*.*	1,151ms	5/24~7/11	33	S ***	HQ Office
	115.94.*.*	1,152ms	6/14	3	BOR ***	PJT Office 1
	106.249.*.*	1,586ms	6/16	1	BOR ***	PJT Office 1
	112.153.*.*	1,233ms	6/19	1	Xsp ***	PJT Office 2
	211.254.*.*	806ms	6/20	1	BOR ***	PJT Office 1
Detected	106.101.*.*	48,933ms	7/13	4	L ** Telco	Hot-spot

**Table 10:** User-agent information of the detected event.

Device	User-Agent Data
Win 10 PC	Mozilla/5.0 (Windows NT 10.0; Win64; x64) AppleWebKit/537.36 (KHTML, like Gecko) Chrome/114.0.0.0 Safari/537.36)

## Data Availability

There is no additional data.

## Appendix A

**Abbreviation**	**Full Name**
RBA	Risk-based authentication
OIDC	OpenID Connect
VPN	Virtual Private Network
MIS	Management Information System
SaaS	Software-as-a-Service
IAM	Identity and Access Management
SSO	Single Sign-On
SAML	Security Assertion Markup Language
SCIM	System for Cross-domain Identity Management
MFA	Multi-Factor Authentication
ISP	Internet Service Provider
IMEI	International Mobile Equipment Identity
RTT	Round-Trip Time
AWS	Amazon Web Services
PING	Packet Internet Groper
JS	Javascript
OAuth	Open Authentication
TXT	Token Exchange Time
GCP	Google Cloud Platform

## Appendix B

A sample query explanation to profile TXT data from logs wso2 carbon log is described as below. The query syntax followed Logpresso [49]:

table duration = 3mon wso2carbon

# Comment: retrieve logs of recent 3months from WSo2 Identity Server
|sort_time
# Comment: sort by time to prepare calculation of TXT time
|search contains(line,”Authorization Request received for user”) or contains(line,”Found Authorization”)
# Comment: filter log which indicates IAM server issuing authorization code (TXT starting point), and receiving authorization code back (TXT finishing point)
|rexfield = line”USERSTORE\/(ser>.*?)\@[a-z]*.company”
|rexfield = line”(Client:|ClientID)(:|)(?<clientId>.*?)\,”
|rexfield = line”DEBUG.*\-(?<message>.*)”
|fields _time,user,clientId,message,line
# Comment: filter out unnecessary fields that the log contains
|serial[evtctxadd topic=gettime key=user maxrows=0 timeout=60s contains(line,”Authorization Request received for user”)or contains(line,”Found Authorization”)
# Comment: calculates TXT serially with 60seconds time window
|evalc dummy=if(contains(line,”Authorization Request received for user”),evtctxsetvar(“gettime”,user,”issue_time”,_time),null)
# Comment: get start time of TXT
|eval diff_time=if(contains(line,”Found Authorization”),abs(datediff(evtctxgetvar(“gettime”,user,”issue_time”),_time,”msec”)),null)]
# Comment: get end time of TXT and subtract start time to calculate TXT
|fields _time,user,clientId,diff_time,line
|search isnotnull(diff_time)
# Comment: filter out unnecessary fields again
|stats avg(diff_time) as avg_diff, min(diff_time) as min_diff, max(diff_time) asmax_diff, count by user, clientId
# Comment: calculate user’s average, min, max TXT per application
|eval avg_diff=round(avg_diff,-1)
# Comment: round TXT
|search count>=5
# Comment: Only profile the user’s TXT per application when there’s more than 5 authentication records for 3 months

## References

[1] Gartner Forecasts of Global Knowledge Workers Will Work Hybrid by the End of 2023. https://www.gartner.com/en/newsroom/press-releases/2023-03-01-gartner-forecasts-39-percent-of-global-knowledge-workers-will-work-hybrid-by-the-end-of-2023.

[2] Kotak J., Habler E., Brodt O., Shabtai A., Elovici Y. (2023). Information Security Threats and Working from Home Culture: Taxonomy, Risk Assessment and Solutions. Sensors.

[3] NIST Special Publication 800-207 Zero Trust Architecture Released August 2020. https://nvlpubs.nist.gov/nistpubs/SpecialPublications/NIST.SP.800-207.pdf.

[4] Department of Defense (DoD) Zero Trust Reference Architecture Version 2.0. July 2022. https://dodcio.defense.gov/Portals/0/Documents/Library/(U)ZT_RA_v2.0(U)_Sep22.pdf.

[5] Parmar V., Sanghvi H.A., Patel R.H., Pandya A.S. A comprehensive study on passwordless authentication. Proceedings of the 2022 International Conference on Sustainable Computing and Data Communication Systems (ICSCDS).

[6] Papaioannou M., Pelekoudas-Oikonomou F., Mantas G., Serrelis E., Rodriguez J., Fengou M.-A. (2023). A Survey on Quantitative Risk Estimation Approaches for Secure and Usable User Authentication on Smartphones. Sensors.

[7] Wiefling S., Lo Iacono L., Dürmuth M. (2019). Is this really you? An empirical study on risk-based authentication applied in the wild. Proceedings of the ICT Systems Security and Privacy Protection: 34th IFIP TC 11 International Conference, SEC 2019.

[8] Wiefling S., Dürmuth M., Lo Iacono L. More than just good passwords? A study on usability and security perceptions of risk-based authentication. Proceedings of the Annual Computer Security Applications Conference.

[9] Bumiller A., Barais O., Aillery N., Le Lan G. Towards a Better Understanding of Impersonation Risks. Proceedings of the 2022 15th International Conference on Security of Information and Networks (SIN).

[10] Doerfler P., Thomas K., Marincenko M., Ranieri J., Jiang Y., Moscicki A., McCoy D. Evaluating login challenges as a defense against account takeover. Proceedings of the World Wide Web Conference.

[11] Andriamilanto N., Allard T., Guelvouit G.L. (2021). “Guess Who?” Large-scale data-centric study of the adequacy of browser fingerprints for web authentication. Proceedings of the Innovative Mobile and Internet Services in Ubiquitous Computing: Proceedings of the 14th International Conference on Innovative Mobile and Internet Services in Ubiquitous Computing (IMIS-2020).

[12] Alaca F., Van Oorschot P.C. Device fingerprinting for augmenting web authentication: Classification and analysis of methods. Proceedings of the 32nd Annual Conference on Computer Security Applications.

[13] Wiefling S., Dürmuth M., Iacono L.L. (2021). What’s in score for website users: A data-driven long-term study on risk-based authentication characteristics. Proceedings of the Financial Cryptography and Data Security: 25th International Conference, FC 2021.

[14] Balebako R., Marsh A., Lin J., Hong J., Cranor L.F. (2014). The privacy and security behaviors of smartphone app developers. Workshop on Usable Security.

[15] Bhuyan S.S., Kim H., Isehunwa O.O., Kumar N., Bhatt J., Wyant D.K., Kedia S., Chang C.F., Dasgupta D. (2017). Privacy and security issues in mobile health: Current research and future directions. Heal. Policy Technol..

[16] Alt F., Schneegass S. (2022). Beyond Passwords—Challenges and Opportunities of Future Authentication. IEEE Secur. Priv..

[17] Acar G., Juarez M., Nikiforakis N., Diaz C., Gürses S., Piessens F., Preneel B. FPDetective: Dusting the web for fingerprinters. Proceedings of the 2013 ACM SIGSAC Conference on Computer & Communications Security.

[18] Zheleva E., Getoor L. (2011). Privacy in social networks: A survey. Social Network Data Analytics.

[19] Madden M., Rainie L. (2015). Americans’ Attitudes about Privacy, Security and Surveillance.

[20] Egelman S., Tsai J., Cranor L.F., Acquisti A. Timing is everything? The effects of timing and placement of online privacy indicators. Proceedings of the SIGCHI Conference on Human Factors in Computing Systems.

[21] Device Fingerprinting and User Privacy: Striking the Right Balance. https://medium.com/@TrustDecision/device-fingerprinting-and-user-privacy-striking-the-right-balance-f67b63e555d9.

[22] Bonneau J., Herley C., van Oorschot P.C., Stajano F. The quest to replace passwords: A framework for comparative evaluation of web authentication schemes. Proceedings of the 2012 IEEE Symposium on Security and Privacy.

[23] Debatin B., Lovejoy J.P., Horn A.K., Hughes B.N. (2009). Facebook and online privacy: Attitudes, behaviors, and unintended consequences. J. Comput. -Mediat. Commun..

[24] Wiefling S., Jørgensen P.R., Thunem S., Iacono L.L. (2022). Pump Up Password Security! Evaluating and Enhancing Risk-Based Authentication on a Real-World Large-Scale Online Service. ACM Trans. Priv. Secur..

[25] Aikat J., Kaur J., Smith F.D., Jeffay K. Variability in TCP round-trip times. Proceedings of the 3rd ACM SIGCOMM Conference on Internet Measurement.

[26] Chen X., Kim H., Aman J.M., Chang W., Lee M., Rexford J. Measuring TCP round-trip time in the data plane. Proceedings of the Workshop on Secure Programmable Network Infrastructure.

[27] Draper-Gil G., Lashkari A.H., Mamun M.S.I., Ghorbani A.A. Characterization of encrypted and vpn traffic using time-related. Proceedings of the 2nd International Conference on Information Systems Security and Privacy (ICISSP).

[28] Lv S., Wang C., Wang Z., Wang S., Wang B., Zhang Y. (2023). AAE-DSVDD: A one-class classification model for VPN traffic identification. Comput. Netw..

[29] Kohls K., Diaz C. {VerLoc}: Verifiable Localization in Decentralized Systems. Proceedings of the 31st USENIX Security Symposium (USENIX Security 22).

[30] Eriksson B., Barford P., Sommers J., Nowak R. (2010). A learning-based approach for IP geolocation. Proceedings of the Passive and Active Measurement: 11th International Conference, PAM 2010.

[31] Kruger C.P., Hancke G.P. Enhanced security in industrial internet of things networks using latency based fingerprinting. Proceedings of the 2020 IEEE 18th International Conference on Industrial Informatics (INDIN).

[32] Ezin L.E.C., Sadre R. Efficient probing of heterogeneous iot networks. Proceedings of the 2017 IFIP/IEEE Symposium on Integrated Network and Service Management (1M).

[33] Aneja S., Aneja N., Islam M.S. Iot device fingerprint using deep learning. Proceedings of the 2018 IEEE International Conference on Internet of Things and Intelligence System (IOTAIS).

[34] Lontorfos G., Fairbanks K.D., Watkins L., Robinson W.H. Remotely inferring device manipulation of industrial control systems via network behavior. Proceedings of the2015 IEEE 40th Local Computer Networks Conference Workshops (LCN Workshops).

[35] Watkins L., Robinson W.H., Beyah R. (2011). A Passive Solution to the CPU Resource Discovery Problem in Cluster Grid Networks. IEEE Trans. Parallel Distrib. Syst..

[36] Watkins L., Robinson W.H., Beyah R. (2015). Using network traffic to infer hardware state: A kernel-level investigation. ACM Trans. Embed. Comput. Syst..

[37] Rivera E., Tengana L., Solano J., Castelblanco A., López C., Ochoa M. Risk-based authentication based on network latency profiling. Proceedings of the 13th ACM Workshop on Artificial Intelligence and Security.

[38] Inspectors Insights—Messages Table. https://docs.telerik.com/fiddler-everywhere/user-guide/inspector-types#messages-tab.

[39] Abdou A., Matrawy A., Van Oorschot P.C. Accurate manipulation of delay-based internet geolocation. Proceedings of the 2017 ACM on Asia Conference on Computer and Communications Security.

[40] Orfanos M., Perakis H., Gikas V., Retscher G., Mpimis T., Spyropoulou I., Papathanasopoulou V. (2023). Testing and Evaluation of Wi-Fi RTT Ranging Technology for Personal Mobility Applications. Sensors.

[41] Garcia-Fernandez M., Hoyas-Ester I., Lopez-Cruces A., Siutkowska M., Banqué-Casanovas X. (2021). Accuracy in WiFi Access Point Position Estimation Using Round Trip Time. Sensors.

[42] Selvan S., Mahinderjit Singh M. (2022). Adaptive contextual risk-based model to tackle confidentiality-based attacks in fog-IoT paradigm. Computers.

[43] Bian H., Bai T., Salahuddin M.A., Limam N., Daya A.A., Boutaba R. (2021). Uncovering Lateral Movement Using Authentication Logs. IEEE Trans. Netw. Serv. Manag..

[44] Department of Defense (DoD) Identity and Access Management Recommended Best Practices for Administrators. March 2023. https://media.defense.gov/2023/Mar/21/2003183448/-1/-1/0/ESF%20IDENTITY%20AND%20ACCESS%20MANAGEMENT%20RECOMMENDED%20BEST%20PRACTICES%20FOR%20ADMINISTRATORS%20PP-23-0248_508C.PDF.

[45] Identity Server Documentation. https://is.docs.wso2.com/en/5.11.0/.

[46] Deploying the Playground2 webapp—Download the Sample. https://is.docs.wso2.com/en/5.11.0/learn/deploying-the-sample-app/#download-the-sample_1.

[47] WHOIS. https://whois.kisa.or.kr.

[48] Salvato M., De Vito S., Guerra S., Buonanno A., Fattoruso G., Di Francia G. An adaptive immune based anomaly detection algorithm for smart WSN deployments. Proceedings of the 2015 XVIII AISEM Annual Conference.

[49] Query Syntax. https://docs.logpresso.com/en/query/query-syntax.

